# The Clinical Features and Diagnosis of Adrenoleukodystrophy: A Case Series of Iranian Family

**Published:** 2016

**Authors:** Parvaneh KARIMZADEH, Narjes JAFARI, Habibe NEJAD BIGLARI, Sayena JABBEHDARI, Mehdi ALIZADEH, Ghazal ALIZADEH, Hamid NEJAD BIGLARI, Sara SANII

**Affiliations:** 1 Pediatric Neurology Research Center, Shahid Beheshti University of Medical Sciences, Tehran, Iran; 2 Pediatric Neurology Center of Excellence, Department of Pediatric Neurology, Mofid Children Hospital, Faculty of Medicine, Shahid Beheshti University of Medical Sciences, Tehran, Iran; 3 Pediatrician, Mahneshan Razi General Hospital, Zanjan Medical University of Medical Sciences, Zanjan, Iran; 4 General Physician, Seyed-o-Shohada NAJA General Hospital, Kerman, Iran; 5 Department of Peri-Natal Medicine and Neonatology, Mofid Children Hospital, Shahid Beheshti University of Medical Sciences, Tehran, Iran; 6 Students’ Research Committee, Faculty of Medicine, Shahid beheshti University of Medical Sciences, Tehran, Iran

**Keywords:** Adrenoleukodystrophy, Neurometabolic disorder, Early detection, Early intervention

## Abstract

**Objective:**

Adrenoleukodystrophy disorder is one of the x-linked genetic disorders caused by the myelin sheath breakdown in the brain. In this study, we present 4 yr experience on this disorder.

**Materials & Methods:**

The patients diagnosed as adrenoleukodystrophy in the Neurology Department of Mofid Children’s Hospital in Tehran, Iran from 2010 to 2014 were enrolled into the study. The disorder was confirmed by neuroimaging and clinical findings along with genetic and neurometabolic assessment at Reference Laboratory in Germany. We assessed age, gender, past medical history, developmental status, clinical manifestations, and neuroimaging findings of populous family with adrenoleukodystrophy.

**Results:**

All of the patients were one populous family with high rate of consanguineous marriages. This disorder was confirmed by genetic assessment, VLCFA and brain MRI. c.253_254insC, p.R85Pfs112* was found in heterozygote state and the VLCFA assessment showed the typical pattern for adrenoleukodystrophy/ adrenomyeloneuropathy. This diagnosis was in agreement with the family history and the clinical history of the patient. Since there have been a number of cases in patient’s family in the past, so intensive follow-up on the family especially detection the female members of the family of childbearing age was recommended. The amount of C-26, C24/C22 and C26/C22 was elevated. All patients with the same genotype had wide ranges of clinical presentation.

**Conclusion:**

Early diagnose of this disease might help us for early intervention and prenatal diagnosis for the disease in next siblings.

## Introduction

Adrenoleukodystrophy is one of the x-linked genetic disorders presented with myelin sheath breakdown in the brain around the nerve cells and progressive adrenal insufficiency (1). It is related to the accumulation of very long chain fatty acids characterized by cerebral demyelination, adrenomyeloneuropathy and myelopathy (2,3). Adrenoleukodystrophy is caused due to the ABCD1 gene defect and resulted to impairment of peroxisomal beta-oxidation. The adrenal cortex, CNS and the leydig cells of testes are the sites of involvement. The ALD phenotypes vary in childhood and adults. Rapidly progressive cerebral disease is seen in childhood form of ALD. Adrenomyeloneuropathy with or without local CNS demyelination is found in adult form of this disorder (4). In this study, we present 4 years of experience on adrenoleukodystrophy from the Pediatric Neurology Research Center of Mofid Children’s Hospital, Tehran, Iran. We describe clinical symptoms and neuroimaging findings for populous family with this disorder.

## Materials & Methods

This observational study was performed on one populous family who lived in Lorestan Province, western Iran from 2010 to 2014. Some children of this family were diagnosed as adrenoleukodystrophy and died owing to this progressive disorder. Whereas the rate of consanguineous marriages in this family was high and some children were presented with adrenoleukodystrophy; all of the family members were undergone genetic assessment. Some of the patients with positive genetic assessment and various presentations of adrenoleukodystrophy such as spasticity, gait disorders, urinary disorders, neurogenic bladder, motor slowing, tremor and non-significant manifestations were undergone Magnetic Resonance Spectoscopy (MRS). The diagnosis was performed based on clinical manifestations, neuroimaging findings, VLCFA, and genetic assessment in referral laboratories in Germany.

**Data analysis**

Data were analyzed using descriptive methods and no statistical testing was applied.

**Ethical approval**

Ethics Committee of Pediatric Neurology Research Center, Shahid Beheshti University of Medical Sciences, Tehran, Iran approved the study. All parents signed a written informed consent for participation in the study.

## Results

Adrenoleukodystrophy disease was found in five boys of one populous family. This disorder approximately was started from the age of 6 yr, with seizure and behavior disorders specially ADHD in addition to progressive motor disorder, ataxia, insomnia etc. All the patients had adrenal insufficiency. In supplementary assessment, leukodystrophy was found in brain MRI, which showed leukodystrophic pattern from posterior to anterior side. Adrenoleukodystrophy was confirmed by VLCFA (very long chain fatty acid) and genetic assessment. Four patients with adrenoleukodystrophy died in the age of 12-13 yr. One patient suffered and was bedridden due to this disorder in the age 24 yr. In this group, ALD presented with gait abnormality, spasticity, clonus, paresthesia, increased deep tendon reflex, positive Romberg, urinary as well as sexual system abnormality and occasional headache. Some patients suffered from urinary reflux and renal failure due to spastic neurogenic bladder. Only one patient had Addison’s disease in his age of 41 yr. He was stricken to Addison’s disease owing to stress of appendicitis surgery and then prednisolone and cortisone were prescribed for his insufficiency. Two years before his admission, nephrologic presentations, writing abnormality and sexuality disorder such as erectile dysfunction started. Sensory motor poly neuropathy was reported in EMG-NCV of group with homozygote men. In another group, women with heterozygote genotype of this mutation were completely sign and symptom free. The oldest one was 67 yr-old woman with normal VLCFA and brain MRI without any symptom. A 25-yr old woman in this group was offspring of consanguineous marriage with gait disorder, spastic extremity, urinary abnormality such as urgent incontinency and spastic bladder presented since her 22 yr of age. Her mother was heterozygote for this mutation. She had a brother with typical ALD, positive VLCFA, confirmed genetic assessment and his brain MRI showed leukodystrophic pattern. Her father was positive for ALD with leukodystrophic pattern in his brain MRI who was bedridden, completely spastic and mild to moderate mental status regression with primary manifestation of adrenomyeloneuropathy in his 34 yr of age. In another family, a 10-yr-old boy died due to ALD. Brain MRI was done because of his headache as a primary manifestation, which showed typical leukodystrophic pattern, and his disorder was confirmed by VLCFA and genetic assessment. He did not have Addison’s disease and his brother with negative genotype for ALD nominated for bone marrow transplantation. The other brother of this boy had similar genotype with confirmed ALD in VLCFA but brain MRI was completely normal. He underwent special diet with Lorenzo oil and did not have any clinical and imaging (Brain MRI) abnormality in his annual assessment for at least 3 yr. c.253_254insC, p.R85Pfs112* was found in heterozygote state by analysis of DNA extraction, analysis of previously detected mutation of ABCD1 gene by PCR and direct sequencing. The above DNA change is a frame shift mutation that makes a truncated 197aa protein in which only the first 85 amino acids have original sequence and text 112 amino acids are changed. The mutation is novel of no previous reported, but certainly is pathogenic. As a sample, the VLCFA of one patient showed the typical pattern for adrenoleukodystrophy/ adrenomyeloneuropathy. This diagnosis was in agreement with the family history and the clinical history of the patient. Since there have been a number of cases in his family in the past, so intensive follow-up on the family especially to have the female members of the family of child-bearing age ready to call, was recommended. The amount of C-26, C24/C22 and C26/C22 was elevated.

## Discussion

Adrenoleukodystrophy is one of the x-linked recessive disorders with variable phenotype and elevated level of long chain fatty acids (5). In our study, we present adrenoleukodystrophy disease in one populous family. However all patients had the same genotype but variable presentations were seen. Even the presentations among the homozygote man were not similar. With the same genotype, ALD presented in one case on his 6 yr of age and occurred in another one on his 10 yr of age. The brain involvement was different between the similar age ranges. Probably, these differences could be justified with either diet variation or environmental factors. The high levels of hexacosansaure (C-26), tetracosansaure (C-24), C24/C22 ratio and C26/C22 ratio are associated with intense and more signs and symptoms. Semmler et el. in their review article done on patients with adrenoleukodystrophy showed that approximately half of the heterozygote female had moderate spastic paresis resembling the phenotype of adrenomyeloneuropathy. They focused on Lorenzo’s oil as a treatment of ALD and reported its positive affection in asymptomatic patients without cerebral involvement and female carriers and VLCFA restriction. It was not effective in cerebral inflammatory disease variants (4). Moser in study that was done on 2000 ALD patients reported the diet of glyceryl trierucate and glyceryl trioleate oil (Lorenzo’s oil) on ALD treatment. Neurological involvement was less severs after treatment with Lorenzo’s oil (6). Brett et al. reviewed the genetic forms of adrenal deficiency and explained the clinical presentation and diagnosis of diseases such as ALD (7). Several papers have been published on the ALD manifestations and CT scan abnormalities (7, 8). Vollkow et al. demonstrated the CT scan, MRI and PET results of patients with ALD, where MRI showed a larger area of abnormality, which was more sensitive in disease detection than CT scan described classical pattern of ALD with less clinical findings relationship (9). Moser et al. reviewed the effect of Lorenzo’s oil on asymptomatic boys with the risk of childhood ALD, which could decrease the rate of adrenomyeloneuropathy progression (2). Silveri et al. (1) reported the symptoms of lower urinary tract among 14 children and adults 8 to 53 yr old with different manifestations. They complained voiding dysfunction the same as some patients in our study.

**In conclusion**, in patients with high number of ALD cases in family, intensive follow-up especially on the female members of the family of childbearing age should be considered and recommended.

## Notes

**Conflicting of interest**: None declared
